# Prevention of non-infectious pulmonary complications after intra-bone marrow stem cell transplantation in mice

**DOI:** 10.1371/journal.pone.0273749

**Published:** 2022-09-09

**Authors:** Yoshiko Yamasuji-Maeda, Hisakazu Nishimori, Keisuke Seike, Akira Yamamoto, Hideaki Fujiwara, Taiga Kuroi, Kyosuke Saeki, Haruko Fujinaga, Sachiyo Okamoto, Ken-ichi Matsuoka, Nobuharu Fujii, Takehiro Tanaka, Masahiro Fujii, Katsumi Mominoki, Takuro Kanekura, Yoshinobu Maeda

**Affiliations:** 1 Department of Hematology and Oncology, Okayama University Graduate School of Medicine, Dentistry and Pharmaceutical Sciences, Okayama, Japan; 2 Department of Dermatology, Kagoshima University Graduate School of Medical and Dental Sciences, Kagoshima, Japan; 3 Department of Transfusion Medicine, Okayama University Hospital, Okayama, Japan; 4 Department of Pathology, Okayama University Graduate School of Medicine, Dentistry and Pharmaceutical Sciences, Okayama, Japan; 5 Department of Animal Resources, Advanced Science Research Center, Okayama University, Okayama, Japan; Indiana University School of Medicine, UNITED STATES

## Abstract

Non-infectious pulmonary complications including idiopathic pneumonia syndrome (IPS) and bronchiolitis obliterans syndrome (BOS), which are clinical and diagnostic manifestations of lung chronic graft-versus-host disease (GVHD), cause significant mortality after allogeneic stem cell transplantation (SCT). Increasing evidence suggests that alloantigen reactions in lung tissue play a central role in the pathogenesis of IPS and BOS; however, the mechanism is not fully understood. Several clinical and experimental studies have reported that intra-bone marrow (IBM)-SCT provides high rates of engraftment and is associated with a low incidence of acute GVHD. In the present study, allogeneic SCT was conducted in mouse models of IPS and BOS, to compare intravenous (IV)-SCT with IBM-SCT. Allogeneic IBM-SCT improved the clinical and pathological outcomes of pulmonary complications compared to those of IV-SCT. The mechanisms underlying the reductions in pulmonary complications in IBM-SCT mice were explored. The infiltrating lung cells were mainly CD11b+ myeloid and CD3+ T cells, in the same proportions as in transplanted donor cells. In an *in vivo* bioluminescence imaging, a higher proportion of injected donor cells was detected in the lung during the early phase (1 h after IV-SCT) than after IBM-SCT (16.7 ± 1.1 vs. 3.1 ± 0.7 × 10^5^ photons/s/animal, IV-SCT vs. IBM-SCT, *P* = 1.90 × 10^−10^). In the late phase (5 days) after SCT, there were also significantly more donor cells in the lung after IV-SCT than after IBM-SCT or allogeneic-SCT (508.5 ± 66.1 vs. 160.1 ± 61.9 × 10^6^ photons/s/animal, IV-SCT vs. IBM-SCT, *P* = 0.001), suggesting that the allogeneic reaction induces sustained donor cell infiltration in the lung during the late phase. These results demonstrated that IBM-SCT is capable of reducing injected donor cells in the lung; IBM-SCT decreases donor cell infiltration. IBM-SCT therefore represents a promising transplantation strategy for reducing pulmonary complications, by suppressing the first step in the pathophysiology of chronic GVHD.

## Introduction

Despite advances in the treatment of graft-versus-host disease (GVHD) and supportive care after hematopoietic stem cell transplantation (SCT), lung manifestations of noninfectious complications, including idiopathic pneumonia syndrome (IPS) and bronchiolitis obliterans syndrome (BOS), continue to confer a poor prognosis [1–4]. IPS refers to a generalized, noninfectious, inflammatory lung injury occurring after SCT [5]. It is characterized by symptoms and signs of pneumonia, a restrictive-type pulmonary function test abnormality, and alveolar injury without lower respiratory tract infection [6]. Several reports have shown that donor T cells are critical for the early proinflammatory events associated with lung injury, while donor lymphocytes continue to respond to host antigens and contribute to physiologically significant lung injury at later time points [7–10]. BOS is the chronic manifestation of GVHD in the lung that results from an immune reaction in the small terminal airways, leading to fibrotic remodeling and occlusion [1]. Although BOS is clinically diagnosed based on pulmonary function tests and high-resolution computed tomography, the pathological diagnosis is called bronchiolitis obliterans (BO) [11]. Increasing evidence suggests that T-cell-mediated recognition of alloantigens expressed in the lung tissue constitutes a central event in the pathogenesis of BOS [12]. However, the pathogenesis of IPS and BOS is not fully understood.

Direct intra-bone marrow (IBM) SCT of cord blood cells, established by Frassoni et al., improves engraftment success and shortens the time until hematological recovery in patients undergoing single cord blood cell transplantation (CBT) [13]. The rationale for intra-bone infusion is based on animal models [14, 15]; in those models, following intravenous (IV) administration, most of the cells were trapped in peripheral organs, with < 10% reaching the bone marrow niche. Several clinical and experimental studies have reported that IBM-SCT ensures a high rate of engraftment and is associated with a low incidence of acute GVHD [16–18]. Therefore, we hypothesized that entrapment of donor cells in peripheral organs contributes to the pathogenesis of GVHD, and that IBM-SCT could ameliorate both IPS and BOS, especially because injected donor cells are first seen in the lung.

In the present study, allogeneic murine SCT was conducted in mouse models of IPS and BOS, to compare IV-SCT with IBM-SCT with respect to the outcomes of pulmonary complications, the kinetics of the transplanted donor cells, and chemokine expression.

## Materials and methods

### Mice

Female C57BL/6 (H-2^b^) mice were purchased from CLEA (Osaka, Japan) and Charles River Japan (Yokohama, Japan). B6D2F1 (H-2^b/d^) and BALB/c (H-2^d^) recipient mice were purchased from Charles River Japan. B10.BR (H-2^k^) mice were purchased from SLC (Hamamatsu, Japan). FVB/N-Tg (β-actin-luc)-Xen (H-2^q^) mice were purchased from Caliper Life Sciences (Hopkinton, MA, USA). Recipient animals (i.e., those used for SCT) were between 10 and 14 weeks of age. Donor animals were between 10 and 30 weeks of age; appropriate age-matched controls were used. The animals were anesthetized or euthanized by isoflurane inhalation. The euthanasia criteria and timing were in accordance with the guidelines for animal experiments of Okayama University. The duration of the experiment, number of animals used, euthanized, and found dead and causes of death are shown in each figure legend. All animal welfare guidelines of the user training program of Okayama University Advanced Science Research Center were followed. All animal protocols were approved by the Institutional Animal Care and Research Advisory Committee of Okayama University Advanced Science Research Center (protocol number OKU-2015059).

### Bone marrow transplantation

Transplants were performed according to previously described standard protocols [19–21]. For the C57BL/6 (H2^b^)→B6D2F1 (H2^b/d^) transfer (acute GVHD model used to study IPS [19]), lethally (12-13-Gy split dose) irradiated B6D2F1 mice were transplanted with 2 × 10^6^ C57BL/6 spleen T cells and 5 × 10^6^ C57BL/6 T cell-depleted bone marrow (TCD-BM) cells. To establish the C57BL/6 (H2^b^)→B10.BR (H2^k^) BOS model, B10.BR recipients were conditioned with cyclophosphamide (Sigma-Aldrich, St. Louis, MO, USA) (120 mg/kg/day i.p. on days –3 and –2) and irradiation (8.3 Gy on day –1) prior to reconstitution with 1 × 10^7^ C57BL/6 TCD-BM cells plus 5×10^4^ splenic T cells, as previously described [20]. To create the luciferase-expressing transgenic FVB/N-Tg (β-actin-luc)-Xen (H-2^q^)→BALB/c (H-2^d^) mouse model, lethally (9-Gy-split dose) irradiated BALB/c mice were transplanted with 2 × 10^6^ FVB/N-Tg spleen T cells and 5 × 10^6^ FVB/N-Tg TCD-BM cells [21]. T cell depletion and purification were performed using anti-CD90 microbeads, a pan-T-cell isolation kit, and the AutoMACS system (Miltenyi Biotec, Auburn, CA, USA) according to the manufacturers’ instructions. Donor cells were injected intravenously (IV-BMT), or into the bone marrow (IBM-BMT) of recipients, on day 0.

### Intra-bone marrow BMT injection

Donor cells were suspended in Cellmatrix collagen gel matrix (Nitta Gelatin, Inc., Yao, Japan), as described previously [22], and cells in 10 μL of Cellmatrix were injected directly into the bone marrow cavity (as also described previously) [23]. Briefly, the mice were anesthetized and the area from the inguinal region to the knee joint was shaved. The tibia was gently drilled through the patellar tendon into the bone marrow cavity using a 26-gauge needle. Donor cells suspended in collagen gel were aspirated into a microsyringe (50 μL; Ito, Shizuoka, Japan), kept briefly at room temperature, and injected into the bone marrow cavity.

### Evaluation of GVHD and survival

Survival after BMT was monitored daily and the acute GVHD status was clinically assessed every 3 days by summing the change scores for weight loss, posture, activity, fur texture, and skin integrity (maximum score = 10), as described previously [24]. Mice were euthanized at humane endpoints in accordance with the user training program of our Institutional Animal Care and Research Advisory Committee. To evaluate chronic GVHD, animals were weighed every 3 days and assessed for skin manifestations of GVHD. The following scoring system was used [25]: healthy appearance, 0; skin lesions with alopecia < 1 cm^2^ in area, 1; skin lesions with alopecia 1–2 cm^2^ in area, 2; and skin lesions with alopecia > 2 cm^2^ in area, 3. In addition, 0.3 points were assigned for skin disease (lesions or scaling) on the ears, tail, or paws. The minimum score was 0 and the maximum score was 3.9. Lung tissue was evaluated according to the presence of periluminal infiltrates (around airways and vessels) and parenchymal pneumonitis (involving the alveoli or interstitium) using a previously described semiquantitative scoring system that considers both the severity and extent of the histopathology [26]. The score was generated by summation of periluminal infiltrate, pneumonitis, and the extent of injury scores (maximum score = 9).

### Analysis of cells infiltrating the lungs

Donor cells were labeled with CellTrace Violet Cell Proliferation Kit (Invitrogen, Carlsbad, CA, USA) and transplanted via IV or IBM injection. One hour after BMT, the lungs were removed and processed into single-cell suspensions. Cells were then exposed to mAbs against CD3 (Phycoerythrin/Cyanine7, Biolegend, Sandiego, CA) T cells, B220 (fluorescein isothiocyanate, Biolegend) B cells and CD11b (allophycocyanin, Biolegend) myeloid cells. CellTrace-labeled T, B, and myeloid cell populations were analyzed using an MACS Quant flow cytometer (Miltenyi Biotec) running FlowJo software (TreeStar, Ashland, OR, USA).

### RT-qPCR

After euthanasia, the right lungs were removed and stored at –80ºC prior to analysis. Samples were pulverized using a CryoPress (Microtech, Chiba, Japan), mixed with 1.5 mL TRIzol (Invitrogen, Carlsbad, CA, USA) and stored at –80ºC. Total RNA (1 μg) was treated with DNaseI (Invitrogen) and reverse-transcribed by first-strand cDNA synthesis using random primers (Promega, Madison, WI, USA). The target cDNA levels were quantified by real-time PCR. The following TaqMan Universal PCR Master Mix and Assay-on-Demand mouse gene-specific fluorescently labeled TaqMan MGB probes were used in conjunction with the ABI StepOnePlus Real-Time PCR System (Applied Biosystems, Waltham, MA, USA): Mm00441242_m1 (MCP-1). The values were normalized to those of GAPDH using the equation dCt = Ct_target_−Ct_GAPDH_.

### Enzyme-linked immunosorbent assay (ELISA)

Lungs were removed and processed into single-cell suspensions. The supernatants were collected and chemokine levels were measured by ELISA (R&D Systems, Minneapolis, MN, USA) according to the manufacturer’s protocol.

### Pulmonary function tests

Pulmonary function tests were performed as described previously [20]. Briefly, Nembutal-anesthetized mice were intubated and ventilated using the FlexiVent system (Scireq, Montreal, Canada). Pulmonary resistance, elastance, and compliance were determined using FlexiVent software (v5.1).

### Assessment of lung fibrosis

Left lung tissues were fixed in ethanol for 12 h, dehydrated, and embedded in paraffin. Each section was cut to a thickness of 4 μm. Masson’s trichrome-stained sections were used for assessment of subepithelial fibrosis, as described previously [27]. Briefly, 2–4 Masson’s trichrome-stained histological preparations of the left lobe, in which the total length of the epithelial basement membrane of the bronchioles was 1.0–2.5 mm (< 200, 200–400, or > 400 μm in diameter), were selected, and the fibrotic area (which stained blue) 50–100 μm beneath the basement membrane (varying depending on the size of the bronchioles) was measured. The mean score of the fibrotic area divided by the basement membrane length in 2–4 preparations from each mouse was calculated, and the mean subepithelial fibrosis score was then derived for each group. The results are expressed as the area of trichrome staining/ bronchiole basement membrane length (in mm).

### *In vivo* and *ex vivo* imaging of transplanted mice

*In vivo* bioluminescence imaging (BLI) was performed as described previously [21, 28] using the IVIS Spectrum system (Caliper Life Sciences). Imaging was conducted at different time points (1, 2, 3, and 6 h, and 1, 2, and 3 days) using a luciferase-expressing transgenic FVB/N (FVB/N luc^+^) (H-2^q^)→BALB/c (H-2^d^) recipient mouse model. For *ex vivo* BLI, mice were injected with luciferin (150 μg/g body weight i.p.) on day 5 after BMT. Five minutes later, the animals were sacrificed. Selected tissues were prepared and imaged for 15 s. Tissue processing was timed to allow incubation for 3 min. The color change represents the light intensity (red, most intense; blue. least intense), which reflects BLI photon emissions and thus the expression level of donor cells. Imaging data were analyzed and quantified using Living Image Software (ver. 3.2; Caliper Life Sciences).

### Statistical analyses

All results are presented as mean ± standard error of the mean (SEM). Group comparisons of GVHD scores were performed with the Mann-Whitney *U* test. Mean weight, BLI signals, and chemokine levels were compared using the unpaired two-tailed Student *t*-test. Pulmonary function was compared by one-way ANOVA with the Tukey post-hoc multiple comparisons test. Lung fibrosis area was compared by one-way ANOVA with the Bonferroni post-hoc multiple comparisons test. Survival data were compared using the Mantel–Cox log-rank test. A *P-*value < 0.05 was considered to indicate statistical significance. Statistical analyses were performed using STATA software (v.12; StataCorp, College Station, TX, USA).

## Results

### The incidence of GVHD was lower after allogeneic IBM-SCT than IV-SCT

**Fig 1:**
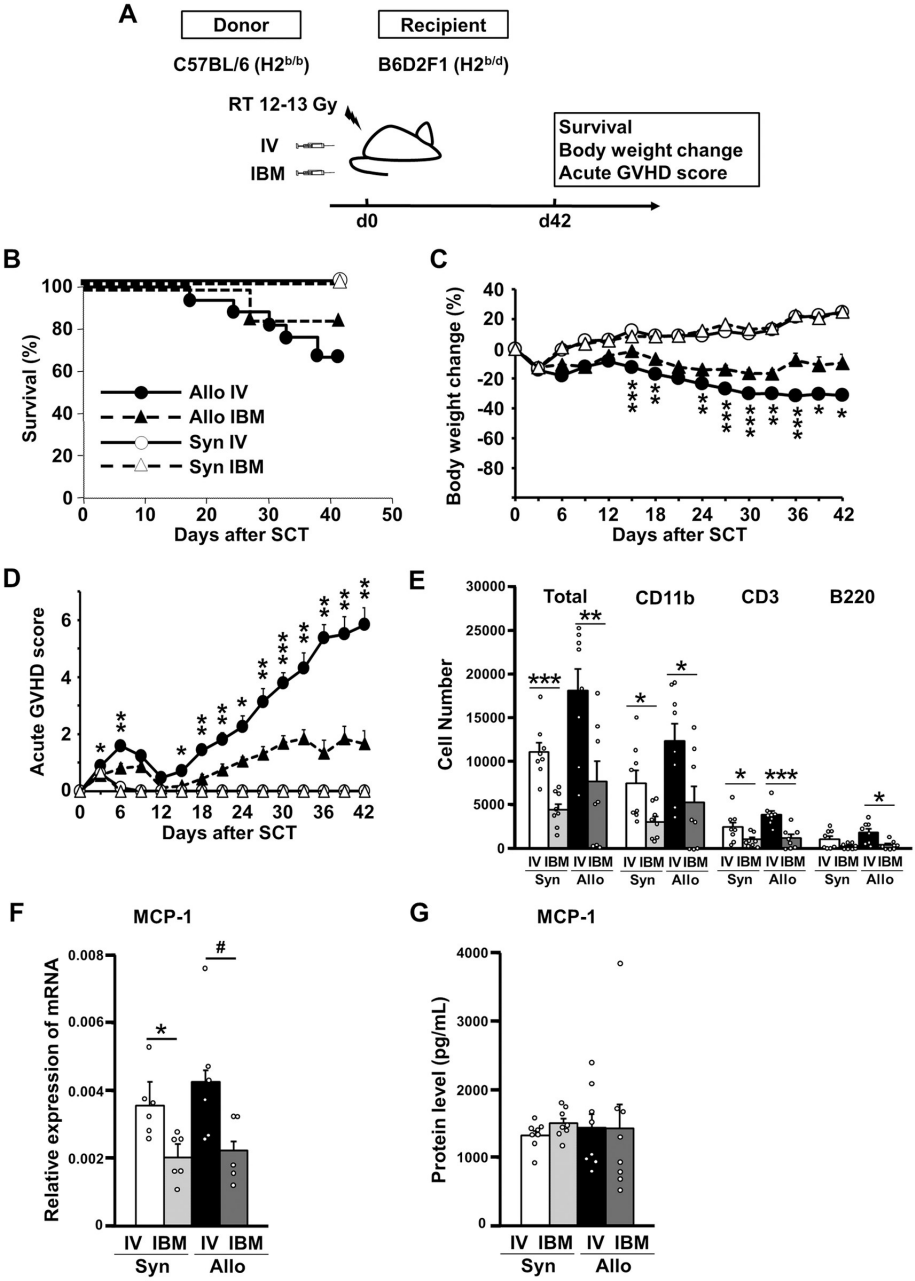
Allogeneic IBM-SCT reduced the incidence of clinical acute GVHD to a greater extent than IV-SCT. (A) Lethally (12–13-Gy split dose) irradiated B6D2F1 mice were administered 2 × 10^6^ C57BL/6 spleen T cells and 5× 10^6^ C57BL/6 TCD-BM cells. The syngeneic group was transplanted with the same doses of TCD-BM cells and splenocytes from B6D2F1 mice. Recipient Kaplan-Meier survival curves were derived; acute GVHD caused death (B), body weight change (C), and an increased acute GVHD score after SCT (D) (Syn IV, *n* = 8; Syn IBM, *n* = 9; Allo IV, *n* = 17; Allo IBM, *n* = 16). Data from two independent experiments were combined. *P < 0.05, **P < 0.01, ***P < 0.001. (E) The transplanted mice (A) were sacrificed 1 h after transplantation and infiltrated cells in the lung were counted (Syn IV, *n* = 8; Syn IBM, *n* = 8; Allo IV, *n* = 8; Allo IBM, *n* = 8). Data from two independent experiments were combined. (F, G) Recipient mice were sacrificed day 2 after transplantation and the levels of mRNAs encoding MCP-1 (F) and the MCP-1 protein levels (G) in the lung were analyzed. (F) (Syn IV, *n* = 6; Syn IBM, *n* = 6, Allo IV; *n* = 6, Allo IBM; *n* = 5) Each graph represents the results of duplicate experiments. (G) (Syn IV, *n* = 8; Syn IBM, *n* = 8, Allo IV; *n* = 8, Allo IBM; *n* = 8), Data from two independent experiments were combined. ^#^ *P* = 0.056, *P < 0.05. GVHD: graft-versus host disease, SCT: stem cell transplantation, Allo: allogeneic, Syn: syngeneic, IV: intravenous, IBM: intra-bone marrow.

Whether the incidence of GVHD is lower following IBM-SCT than after conventional IV-SCT was assessed using a well-characterized experimental mouse model of GVHD, C57BL/6 (H2^b^) → B6D2F1 (H2^b/d^) (Fig 1A) [19]. IBM-SCT recipients had slightly better survival than IV-SCT allogeneic recipients, although there was no statistically significant difference (*P* = 0.398, Fig 1B). As previously demonstrated [17], IBM-SCT recipients showed significantly less weight change (−31.2 ± 2.6 vs. −9.5 ± 5.8%, IV-SCT vs. IBM-SCT at day 42, *P* = 0.012, Fig 1C) and a lower acute GVHD score (5.83 ± 0.60 vs. 1.67 ± 0.45, IV-SCT vs. IBM-SCT at day 42, *P* = 0.006, Fig 1D) than IV-SCT recipients.

Because the injected cells would initially be trapped in the lung, we measured the numbers of various subsets of donor cells infiltrating the lung 1 h after BMT. More donor cells infiltrated after IV-SCT than IBM-SCT in both the syngeneic and allogeneic SCT groups (syngeneic, 11.1 ± 1.0 vs. 4.44 ± 0.7 × 10^3^, *P* = 1.85 × 10^−4^, allogeneic, 18.1 ± 2.4 vs. 7.69 ± 0.2 × 10^3^, *P* = 0.007, respectively, Fig 1E). The increase in total cell number was mainly attributable to an increased number of CD11b+ myeloid cells, followed by CD3+ T cells and B220+ B cells, reflecting the proportions of these cells in the transplanted donor cells (Fig 1E). Evaluation of pulmonary GVHD status on day 42 revealed no significant difference in the extent of pathological lung injuries between IV-SCT and IBM-SCT mice (4.11 ± 0.46 vs. 3.63 ± 0.35, *P* = 0.448), because this mouse model features relatively modest lung injury. High levels of monocyte chemoattractant protein-1 (MCP-1) were associated with BO pathogenesis [29]. We thus measured MCP-1 expression levels in mice with pulmonary GVHD. The lung MCP-1 mRNA expression on day 2 was somewhat higher after allogeneic IV-SCT than allogeneic IBM-SCT (MCP-1, 0.0043 ± 0.00069 vs. 0.0022 ± 0.00038 compared to GAPDH; *P* = 0.056) (Fig 1F). A similar difference was observed between mice that underwent syngeneic IV-SCT and syngeneic IBM-SCT (MCP-1, 0.0036 ± 0.00035 vs. 0.0020 ± 0.00026 compared to GAPDH; *P* = 0.010), suggesting that increases in chemokine levels in the lung during early post-SCT are a characteristic response to SCT injection method. The MCP-1 protein levels did not differ significantly not only between IV-SCT and IBM-SCT, but also between syngeneic SCT and allogeneic SCT (Fig 1G).

**Fig 2:**
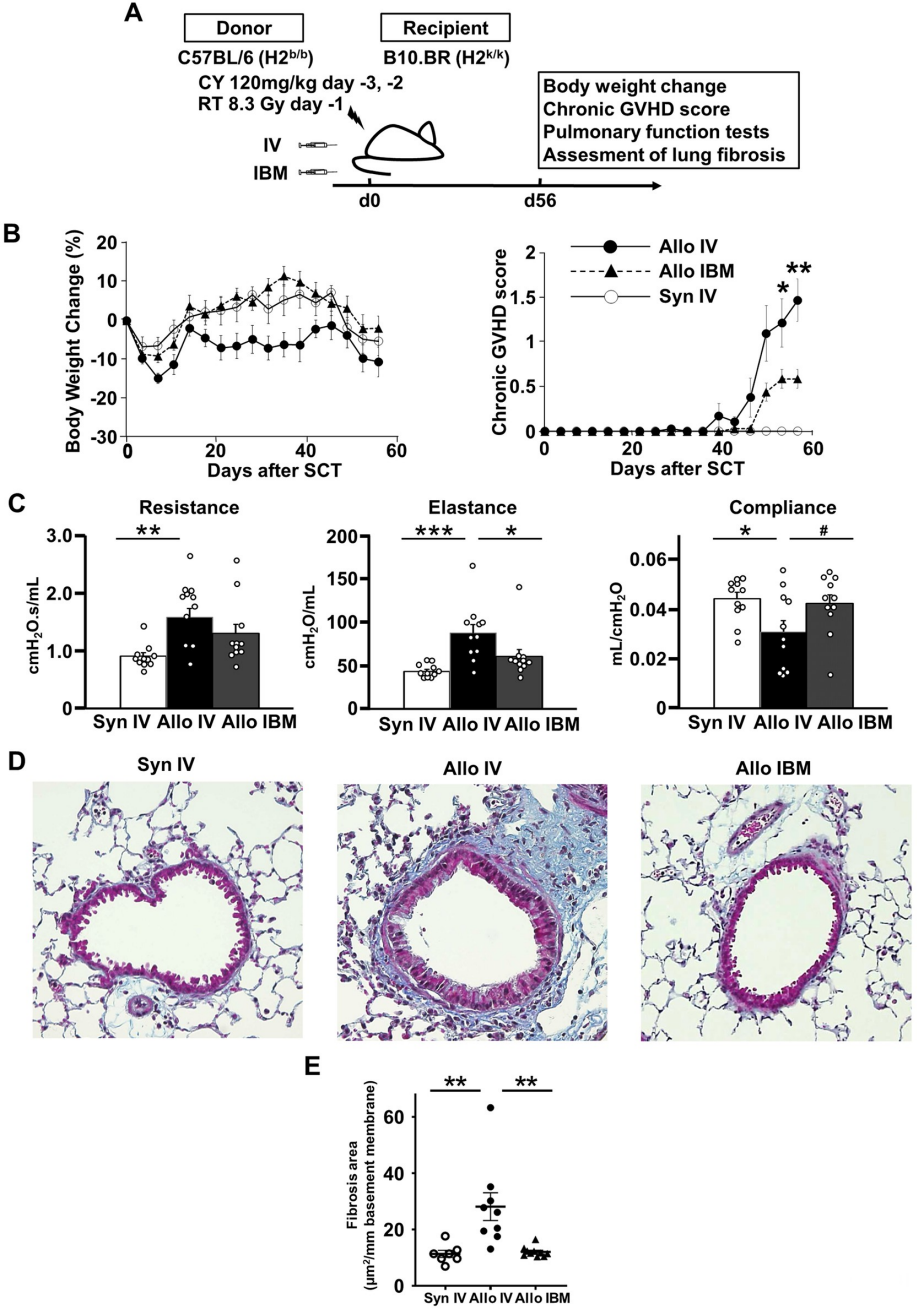
Allogeneic IBM-SCT improved BOS compared to IV-SCT. (A) B10.BR recipients were conditioned with cyclophosphamide (120 mg/kg/day i.p., on days –3 and –2) and irradiation (8.3 Gy, day –1) prior to reconstitution with 1 × 10^7^ C57BL/6 TCD-BM plus 5×10^4^ splenic T cells. (B) The recipients were analyzed in terms of body weight change and chronic GVHD score after SCT (Syn IV, *n* = 6; Allo IV, *n* = 14; Allo IBM, *n* = 10). Data from two independent experiments were combined. *P < 0.05, **P < 0.01. (C) Pulmonary function test data obtained on day 56 post-SCT (Syn IV, *n* = 11; Allo IV, *n* = 11; Allo IBM, *n* = 11). Data from three independent experiments were combined. # *P* = 0.105, *P < 0.05, **P < 0.01, ***P < 0.001. (D) Masson’s trichrome lung tissue staining data obtained 56 days after SCT (representative pictures). (E) Masson’s trichrome-positive fibrotic area, calculated as described in Materials and Methods (Syn IV, *n* = 7; Allo IV, *n* = 9; Allo IBM, *n* = 10). Data from two independent experiments were combined. **P < 0.01. GVHD: graft-versus host disease, SCT: stem cell transplantation, Allo: allogeneic, Syn: syngeneic, IV: intravenous, IBM: intra-bone marrow.

Another mouse model was tested, in which the lung injury was severe enough to determine whether IBM-SCT improves pulmonary GVHD (Fig 2A) [20]. IBM-SCT recipients showed less weight change than IV-SCT recipients, but the difference was not statistically significant (Fig 2B). IBM-SCT recipients had a significantly lower chronic GVHD score than IV-SCT recipients at day 56 (0.6 ± 0.1 vs. 1.5 ± 0.2, *P* = 0.004; Fig 2B). Moreover, clinical BOS manifestations improved in IBM-SCT recipients, including a decrease in pulmonary elastance (87.4 ± 9.6 vs. 60.3 ± 8.1 cmH_2_O/mL, IV-SCT vs. IBM-SCT at day 56, *P* = 0.048) compared to IV-SCT recipients (Fig 2C). Pulmonary resistance and compliance improved slightly, although there were no statistically significant differences (resistance: 1.57 ± 0.15 vs. 1.30 ± 0.16 cmH_2_O.s/mL, IV-SCT vs. IBM-SCT at day 56, *P* = 0.344, compliance: 0.031 ± 0.005 vs. 0.042 ± 0.004 mL/cmH_2_O, IV-SCT vs. IBM-SCT at day 56, *P* = 0.105) (Fig 2C). Histopathological examination of the lung tissues of both groups at 56 days post-SCT showed less fibrotic changes in allogeneic IBM-SCT mice than in allogeneic IV-SCT mice (28.2 ± 4.7 vs. 12.1 ± 0.5 μm^2^/mm basement membrane, IV-SCT vs. IBM-SCT, *P* = 0.002, Fig 2D and 2E). The lung MCP-1 mRNA expression and protein levels on day 2 had no significant differences between the groups (Syngeneic IV-SCT, Syngeneic IBM-SCT, Allogeneic IV-SCT, and Allogeneic IBM-SCT) (S1 Fig). Taken together, these findings demonstrate that allogeneic IBM-SCT improves both clinical BOS, as determined by clinical and histopathologic scores, and pulmonary function compared to IV-SCT.

### Increased number of donor cells in the lungs of IV-SCT mice compared to IBM-SCT mice

**Fig 3:**
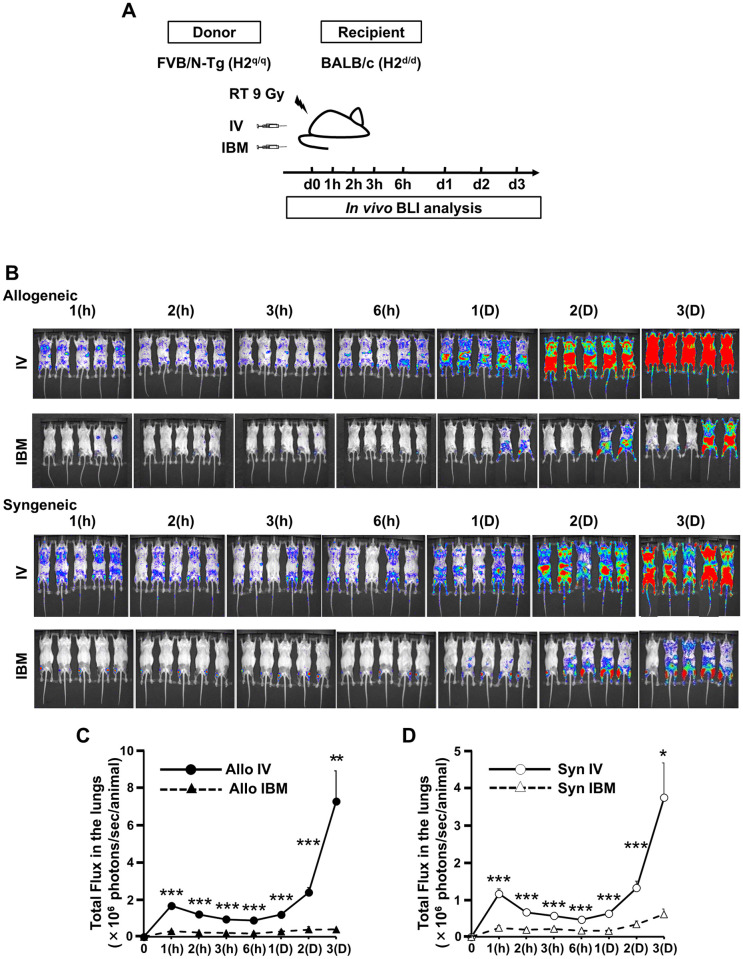
Injected donor cell levels in the lung soon after IV-SCT. (A). Lethally (9-Gy-split dose) irradiated BALB/c mice were transplanted with 2 × 10^6^ FVB/N-Tg spleen T cells and 5 × 10^6^ FVB/N-Tg BM cells, and bioluminescence *in vivo* (BLI) images were obtained at different times (1, 2, 3, and 6 h, and 1, 2, and 3 days) thereafter. (B). BLI images taken at various time points. The color change (red, most intense; blue, least intense) reflects BLI photon emission, and thus the numbers of donor cells. A representative image is shown. (C, D), Quantitative BLI photon emission in the lungs over time. The position of both lungs of the mice were gated manually in each mouse using the elliptical description of the analysis software. (C) After allogeneic SCT (Allo IV, *n* = 18; Allo IBM, *n* = 13) (data from four independent experiments were combined; **P < 0.01, ***P < 0.001) (D) After syngeneic SCT (Syn IV, *n* = 10; Syn IBM, *n* = 10) (data from two independent experiments were combined; *P < 0.05, ***P < 0.001). Allo: allogeneic, Syn: syngeneic, IV: intravenous, IBM: intra-bone marrow, h: hour, D: day.

The mechanism by which IBM-SCT ameliorates pulmonary GVHD was investigated by examining donor cell kinetics in the transplant recipients. We first monitored the *in vivo* distributions of the infused donor cells and compared them between IBM-SCT and IV-SCT mice. The cells were visualized using FVB/N-Tg (β-actin-luc)-Xen (H-2^q^) mice as the donors [21] (Fig 3A), and imaging the recipient mice at different time points (1, 2, 3, and 6 h, and 1, 2, and 3 days). After allogeneic SCT, *in vivo* BLI revealed a higher proportion of donor cells in the lung 1 h after IV-SCT, whereas almost all donor cells were localized in the injected limbs 1 h after IBM-SCT (Fig 3B) and significantly fewer cells had reached the lung (IV-SCT vs. IBM-SCT, 16.7 ± 1.1 vs. 3.1 ± 0.7 × 10^5^ photons/s/animal, *P* = 1.90 × 10^−10^, Fig 3C). After syngeneic (FVB/N luc^+^ into WT FVB/N) SCT, a higher proportion of the injected cells was also detected in the lung 1 h after IV-SCT (Fig 3B). A similar difference was observed in donor cell distribution in the lung after IV-SCT compared to IBM-SCT (11.6 ± 1.3 vs. 2.4 ± 0.6 × 10^5^ photons/s/animal, *P* = 2.13 × 10^−5^; Fig 3D). In this model, allogeneic IBM-SCT recipients had better survival (*P* = 0.014, S2A Fig), less weight change (S2B Fig), a lower acute GVHD score (S2C Fig) and a lower pathological lung GVHD score at day 5 (3.50 ± 0.43 vs. 1.60 ± 0.45, *P* = 0.012, S2D Fig) than those of IV-SCT recipients.

**Fig 4:**
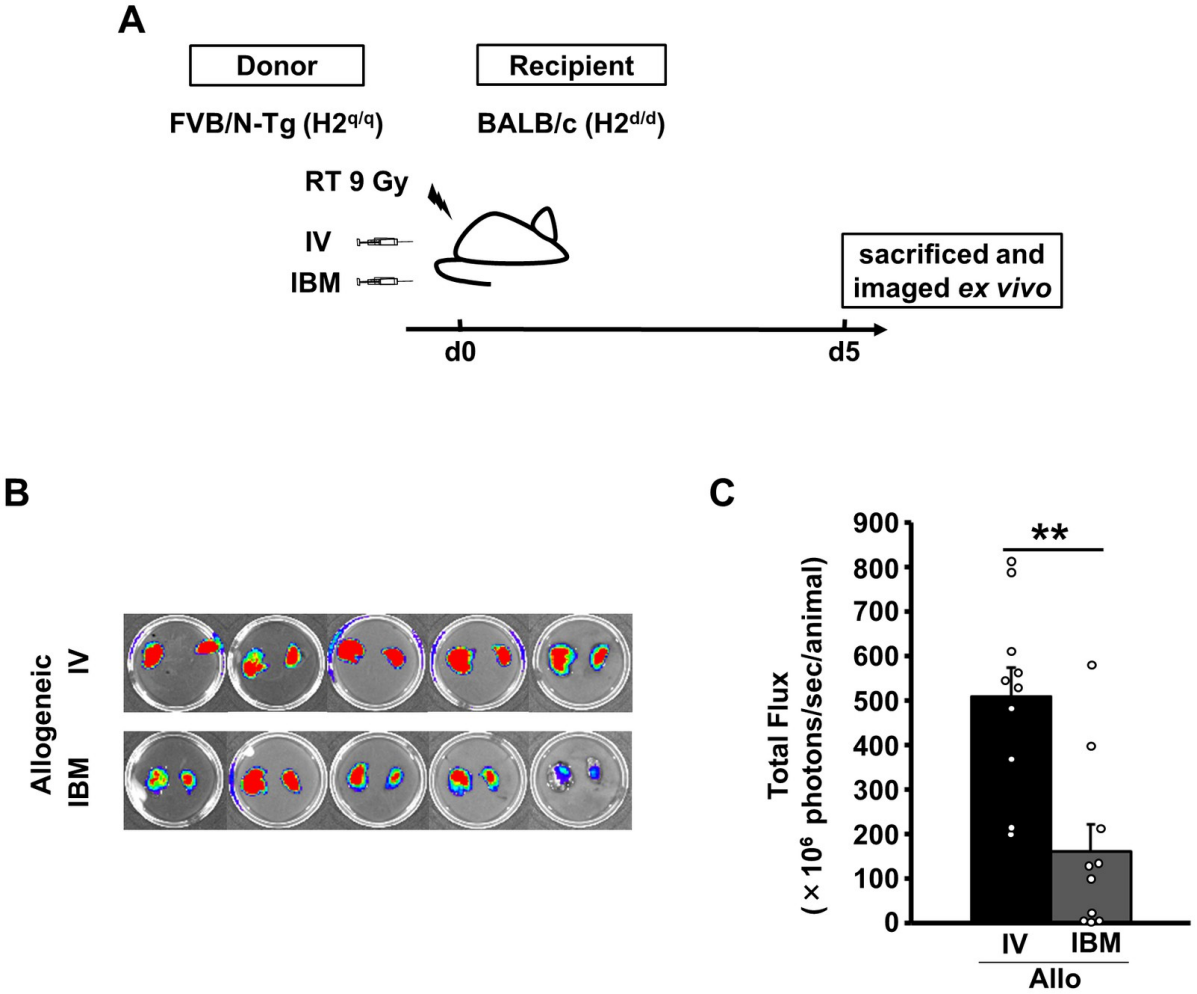
Allogeneic reaction induced sustained donor cell infiltration into the lung. (A) Lethally (9-Gy-split dose) irradiated BALB/c mice were transplanted with 2 × 10^6^ FVB/N-Tg spleen T cells and 5 × 10^6^ FVB/N-Tg BM cells. Recipient mice were sacrificed after 5 days and imaged *ex vivo*. The color change (red, most intense; blue, least intense) reflects donor cell numbers. (B) *Ex vivo* lung images obtained 5 days after transplantation. The color changes (red, most intense; blue, least intense) reflect BLI photon emission, and thus the numbers of donor cells. A representative image is shown. (C) Quantitative *ex vivo* BLI photon emission analysis on day 5 (Allo IV, *n* = 10; Allo IBM, *n* = 10). Data from two independent experiments were combined. **P = 0.001. Allo: allogeneic, IV: intravenous, IBM: intra-bone marrow.

To examine the detailed lung distribution in the later phase, mice from each experimental group were euthanized and the lungs were prepared for *ex vivo* analyses (Fig 4A). On day 5, there were significantly more donor cells in the late phase after IV-SCT than IBM-SCT with allogeneic-SCT (IV-SCT vs. IBM-SCT, 508.5 ± 66.1 vs. 160.1 ± 61.9 × 10^6^ photons/sec/animal, *P* = 0.001, Fig 4B and 4C). These results suggested that the initial cell localization to the lung is dependent on the SCT method, and that a higher proportion of injected donor cells are present in the lung early after IV-SCT. Subsequent allo-reactive responses, which likely amplified the differences in bioluminescence signals of the donor cells between IBM-SCT and IV-SCT mice, may have caused pulmonary complications.

## Discussion

This study showed that most of the transplanted cells remained in the infusion site after IBM-SCT, whereas after IV-SCT the injected donor cells became trapped in the lung. There were significantly fewer donor cells in the late phase after allogeneic IBM-SCT than IV-SCT, which was associated with less severe pulmonary complications in IBM-SCT than IV-SCT recipient mice.

Rocha et al. reported a significantly lower cumulative incidence of acute GVHD following IBM-CBT (19%) than IV-CBT (47%, *P* < 0.0001). According to multivariate analysis, IBM-CBT recipients had a lower incidence of, and less severe, acute GVHD (HR, 0.31; 95% CI, 0.16–0.62; *P* < 0.0008) than IV-CBT recipients [16]. In line with this clinical finding, the present study showed that acute GVHD was significantly suppressed in IBM-SCT mice compared to IV-SCT mice (Fig 1A–1D). We also detected a higher number of donor cells, mainly myeloid cells, and T cells, were infiltrated after IV-SCT compared to IBM-SCT in both syngeneic and allogeneic SCT (Fig 1E). In contrast, almost all donor cells were localized in the injected limbs 1 h after IBM-SCT (Fig 3B). A possible explanation provided by a previous study is that only a portion of transplanted T cells reaches the lymphatic organs, where they would be immediately confronted by host antigen-presenting cells, as probably occurs after IV-SCT [16]. Another possible mechanism is that injected T cells immediately come into contact with mesenchymal stem cells and osteoblasts in the marrow niches, which results in an immunosuppressive effect in IBM-SCT [16, 30–32].

To investigate the mechanism, we focused on MCP-1 expression because several reports showed an association between MCP-1 and BO, IPS, and chronic GVHD [29, 33]. MCP-1 mRNA expression in the lungs was higher in IV-SCT than in IBM-SCT mice of acute GVHD model (Fig 1E). However, there was no significant difference in the protein levels of MCP-1 between not only IV-SCT and IBM-SCT but also between syngeneic and allogeneic SCTs in acute GVHD model, suggesting that the increase in chemokines at the protein level might not exceed the effect of the transplantation invasion itself. In the BO model, the lung MCP-1 mRNA expression and protein levels on day 2 had no significant differences between the groups (Syngeneic IV-SCT, Syngeneic IBM-SCT, Allogeneic IV-SCT, and Allogeneic IBM-SCT) (S1 Fig). This BO model is predominantly fibrosis, mainly in macrophages, and has a marked GVHD score, but is less likely to show differences in cytokines. Mortari et al. demonstrated paradoxically, these mediators were expressed at higher levels at the early Day 7 post-BMT time point in mice given the lower dose of T cells and that go on to develop BO compared with the mice given high dose T cells [34]. In contrast, C57BL/6→B6D2F1 mouse model is a systemic, T-cell-injury model, which might account for this difference of MCP-1 expression. Taken together, we are not able to strongly conclude that MCP-1 is one of the possible mechanisms of GVHD reduction in IBM-SCT.

The detailed pathogenesis of BOS is still not fully understood; however, it is generally considered that the primary event of BOS is T cell-mediated recognition of alloantigens expressed in lung tissue [12]. In bone marrow, antigen presenting cells were immature [35], and the bone marrow dendritic cells migrate through the blood to lymphoid tissues, where they and their progeny divide to fill the dendritic cell compartment [36]. Therefore, mature antigen presenting cells in the bone marrow are fewer than these in the lungs, which could lead to an advantage of IBM-SCT. IBM-SCT may suppress the first step of early inflammation by decreasing the amount of contact with antigen-presenting cells, resulting in reduced exacerbation during the early phase of pulmonary complication.

There are several limitations in this study. In the chemokine analysis, we have not been able to measure the MCP-1 in time series. Further analysis of cellular dynamics and associated the MCP-1 dynamics in mice is needed to elucidate the detailed involvement. Next, it is not clear from this study which cells produce the MCP-1. Moreover, we have not been able to consistently obtain the same results in different mouse models. Further investigation is warranted to elucidate the detailed mechanism of GVHD reduction in IBM-SCT.

In conclusion, this study showed that IBM-SCT reduces pulmonary complication by suppressing the first step in the pathophysiology of chronic GVHD. Further clinical studies are needed to confirm the effectiveness of IBM-SCT; however, IBM-SCT may be an effective strategy for the prevention of pulmonary complication.

## Supporting information

S1 Fig MCP-1 expression in BOS mouse model. B10.BR recipients were conditioned with cyclophosphamide (120 mg/kg/day i.p., on days –3 and –2) and irradiation (8.3 Gy, day –1) prior to reconstitution with 1 × 10^7^ C57BL/6 TCD-BM plus 5×10^4^ splenic T cells. Recipient mice were sacrificed day 2 after transplantation and the levels of mRNAs encoding MCP-1 (A) and the MCP-1 protein levels (B) in the lung were analyzed. (A) (Syn IV, *n* = 8; Syn IBM, *n* = 8, Allo IV; *n* = 10, Allo IBM; *n* = 10) Data from two independent experiments were combined. (B) (Syn IV, *n* = 8; Syn IBM, *n* = 8, Allo IV; *n* = 8, Allo IBM; *n* = 8), Data from two independent experiments were combined.
(TIF)

S2 Fig Allogeneic IBM-SCT reduced acute GVHD compared to IV-SCT in the FVB/N-Tg into BALB/c mouse model. Lethally (9-Gy-split dose) irradiated BALB/c mice were transplanted with 2 × 10^6^ FVB/N-Tg spleen T cells and 5 × 10^6^ FVB/N-Tg BM cells. The recipients were analyzed in terms of survival (A), body weight change (B), and acute GVHD score after SCT (C) (Syn IV, *n* = 10; Syn IBM, *n* = 10; Allo IV, *n* = 10; Allo IBM, *n* = 10). Data from two independent experiments were combined. *P < 0.05. (D) Pathological lung GVHD score obtained on day 5 post-SCT (Allo IV, *n* = 10; Allo IBM, *n* = 10). Data from two independent experiments were combined. *P < 0.05.
(TIF)

S1 Checklist The ARRIVE guidelines 2.0: Author checklist. (PDF)
