# Supplementary figures and images for: Prevention of non-infectious pulmonary complications after intra-bone marrow stem cell transplantation in mice
Source: PLoS One. 2022 Sep 9;17(9):e0273749. doi: 10.1371/journal.pone.0273749 (PMC9462704; doi:10.1371/journal.pone.0273749)

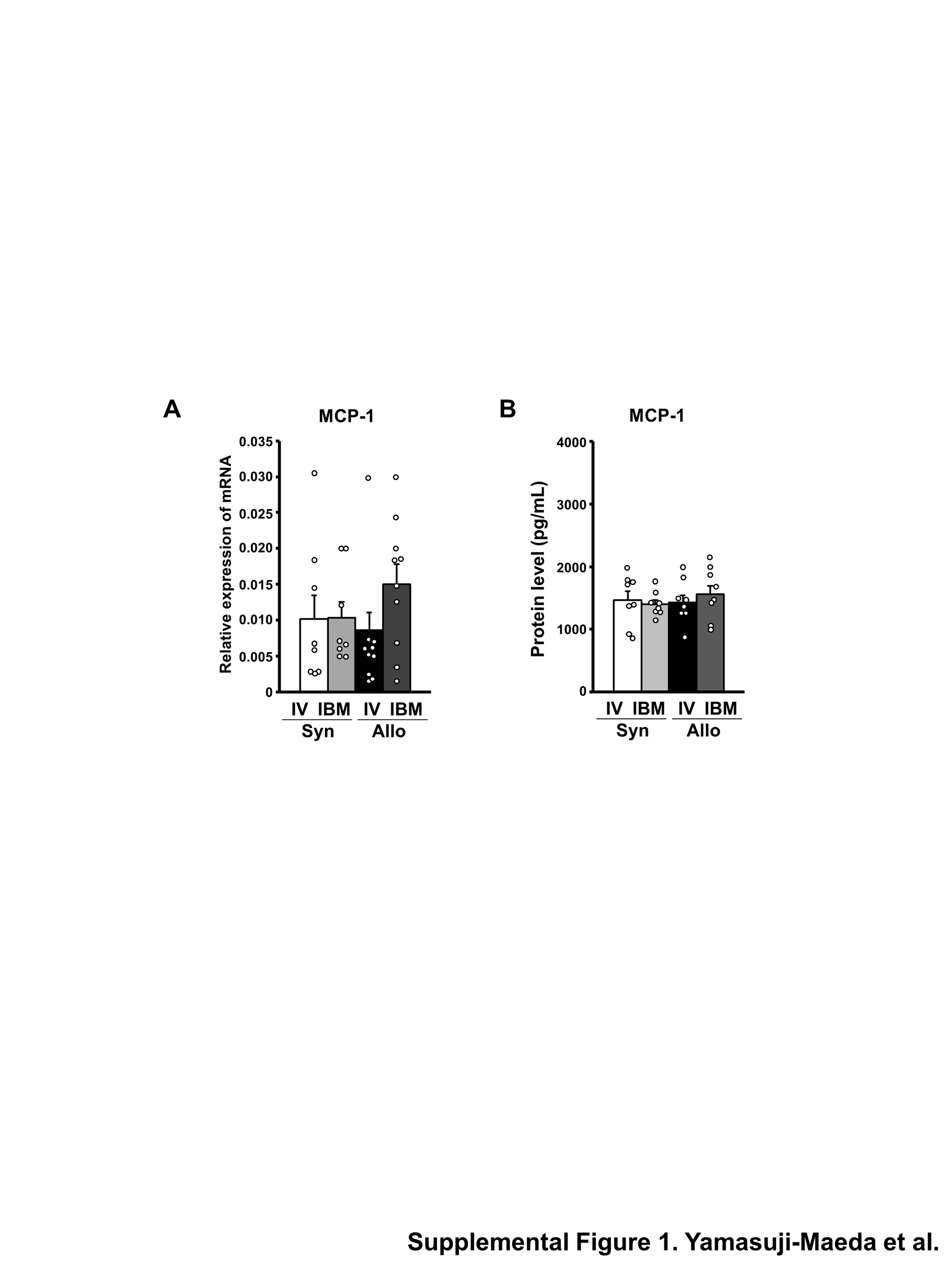

Supplement: S1 Fig — B10.BR recipients were conditioned with cyclophosphamide (120 mg/kg/day i.p., on days –3 and –2) and irradiation (8.3 Gy, day –1) prior to reconstitution with 1 × 10^7^ C57BL/6 TCD-BM plus 5×10^4^ splenic T cells. Recipient mice were sacrificed day 2 after transplantation and the levels of mRNAs encoding MCP-1 (A) and the MCP-1 protein levels (B) in the lung were analyzed. (A) (Syn IV, n = 8; Syn IBM, n = 8, Allo IV; n = 10, Allo IBM; n = 10) Data from two independent experiments were combined. (B) (Syn IV, n = 8; Syn IBM, n = 8, Allo IV; n = 8, Allo IBM; n = 8), Data from two independent experiments were combined. (TIF) [file pone.0273749.s001.tif]

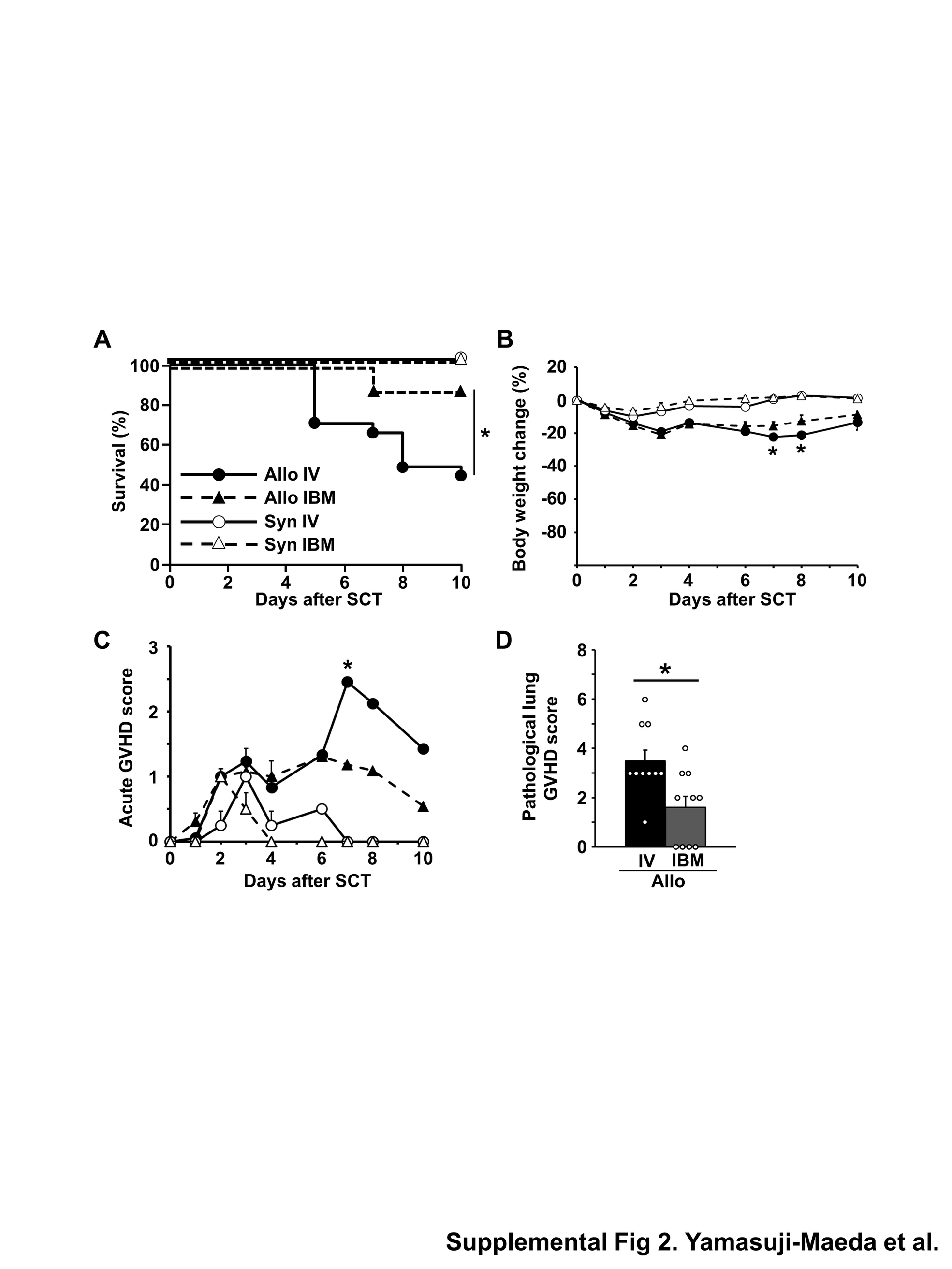

Supplement: S2 Fig — Lethally (9-Gy-split dose) irradiated BALB/c mice were transplanted with 2 × 10^6^ FVB/N-Tg spleen T cells and 5 × 10^6^ FVB/N-Tg BM cells. The recipients were analyzed in terms of survival (A), body weight change (B), and acute GVHD score after SCT (C) (Syn IV, n = 10; Syn IBM, n = 10; Allo IV, n = 10; Allo IBM, n = 10). Data from two independent experiments were combined. *P < 0.05. (D) Pathological lung GVHD score obtained on day 5 post-SCT (Allo IV, n = 10; Allo IBM, n = 10). Data from two independent experiments were combined. *P < 0.05. (TIF) [file pone.0273749.s002.tif]
